# Patient deaths during the period of prolonged stay in cases of delayed discharge for nonclinical reasons at a university hospital: a cross sectional study

**DOI:** 10.7717/peerj.13596

**Published:** 2022-06-17

**Authors:** Amada Pellico-López, Manuel Herrero-Montes, David Cantarero Prieto, Ana Fernández-Feito, Joaquin Cayon-De las Cuevas, Paula Parás-Bravo, María Paz-Zulueta

**Affiliations:** 1 Departamento de Enfermería, Universidad de Cantabria, Santander, Spain; 2 Cantabria Health Service, Santander, Spain; 3 IDIVAL, Grupo de Investigación en Enfermería, Santander, Spain; 4 IDIVAL, Research Group of Health Economics and Health Services Management–Research Institute Marqués de Valdecilla, Santander, Spain; 5 Departamento de Economía, Universidad de Cantabria, Santander, Spain; 6 Facultad de Medicina y Ciencias de la Salud, Departamento de Medicina, Universidad de Oviedo, Oviedo, Spain; 7 Instituto de Investigación Sanitaria del Principado de Asturias (ISPA), Área de Investigación en Cuidados, Grupo de Procesos Asistenciales de Enfermería, Oviedo, Spain; 8 IDIVAL, Grupo de Investigación en Derecho Sanitario y Bioética, GRIDES, Santander, Spain; 9 Departamento de Derecho Privado, Universidad de Cantabria, Santander, Spain

**Keywords:** Length of stay, Patient discharge, Mortality, Palliative care, Patient transfer

## Abstract

**Background:**

Delayed discharge for non-clinical reasons also affects patients in need of palliative care. Moreover, the number of people dying in hospitals has been increasing in recent years. Our aim was to describe characteristics of patients who died during prolonged stay, in comparison with the rest of patients with delayed discharge, in terms of length of hospital stay, patient characteristics and the context of care.

**Methods:**

A descriptive cross-sectional study at a high complexity public hospital in Northern Spain (2007–2015) was conducted. To compare the differential characteristics of the groups of patients died during delayed discharge with the rest, Student’s T test and Pearson’s chi-square test (*χ*^2^) were used.

**Results:**

A total of 198 patients died (6.57% of the total), with a mean total stay of 27.45 days and a prolonged stay of 10.69 days. Mean age 77.27 years. These were highly complex cases, 77.79% resided in the urban area, were admitted urgently (95.45%), to internal medicine or oncology wards, and the most common diagnosis was pneumonia. In people with terminal illness, clinicians can better identify when therapeutic possibilities are exhausted and acute hospitalization is not an adequate resource for their needs. Living in an urban area with the availability of palliative care hospital beds is related to the decision to die in hospital.

## Introduction

Delayed discharge for non-clinical reasons or bed blocking (BB) refers to the period of prolonged stay once the patient is considered clinically stable for discharge from the hospital but remains admitted for non-medical reasons (Rojas-García et al., 2020). This phenomenon is associated with older age (Challis et al., 2014; McCloskey et al., 2014; Gaughan et al., 2017; Ou et al., 2019), pathologies of greater clinical complexity (Gaughan et al., 2017; Holmas, Islam & Kjerstad, 2013), loss of functional capacity (Challis et al., 2014; Gaughan et al., 2017), urgent admissions (McCloskey et al., 2014), surgery, patients requiring subsequent rehabilitation or being referred to a residential care facility for dependent persons upon discharge (Challis et al., 2014). At the family level, the lack of a primary caregiver (Landeiro, Leal & Gray, 2016) or a weak social support network are influential factors (Thomas & Ramcharam, 2010).

The prevalence of this problem varies considerably depending on the context and appears in any type of healthcare system. Thus, BB leads to a cascade of negative effects besides the inefficient and inappropriate use of the acute hospital bed. These include iatrogenic complications due to the increased length of stay, and the negative emotional impact this may have on both patients and family members (Rojas-García et al., 2020).

In the literature there is evidence that situations of BB also arise in patients in need of palliative care (Thomas & Ramcharam, 2010; Soares et al., 2018). The main users of hospital services are those suffering from chronic disease. In the early phases, patients living with chronic diseases may require intermittent treatment in acute hospitalization services for episodes of exacerbation, such as heart failure. However, often advanced disease may represent a terminal event and interventions towards the end of life may be more than standard for a particular illness. Professionals responsible for patient care need to explore the preferences of both the patient and family for this terminal stage. Despite home being the preferred place of death, in recent years, the number of people dying in hospitals has been increasing, because of inadequate symptom control or rapid and unexpected deterioration, which is difficult to manage for both patients and families (Willard & Luker, 2006; Jiménez-Puente & García Alegría, 2018).

It is worthwhile to determine the differential characteristics of cases in which patients have died, compared with other patients with BB, who live in the community and are hospitalized for an acute problem that causes dependence and an inefficient use of health resources. It is necessary to know what is distinctive about these deceased cases compared to other BB cases, in terms of characteristics such as sex, age, length of hospital stay and the relation between stay and external variables, such as destination at discharge.

At present, the coronavirus pandemic increases the need for an efficient use of the hospitalization ward and may cause complicated pneumonias that can affect the most fragile patients, in most cases, older people who are more likely to die. Therefore, it is important to identify cases of people who die while waiting for a more appropriate resource for their situation rather than a conventional acute hospitalization bed. This study sought to describe the characteristics of patients who died during their prolonged stay and to compare them with the remaining patients in a BB situation.

## Materials & Methods

### Study location and population

This study was a descriptive, observational, cross-sectional study based on the analysis of the Hospital Records of Discharged Patients of cases of delayed discharge for non-clinical reasons recorded by the hospital admission service between January 1, 2007 and December 31, 2015.

The setting of this study was the Valdecilla Hospital located in Cantabria (Northern Spain). This high-complexity public hospital has 903 hospitalization beds with high healthcare and technological qualifications. A population of 319,751 people is served by this hospital, which is a hospital of reference for two other local hospitals with a catchment area population of 255,000 people.

The study included all those patients identified as clinically fit for medical discharge by the hospital’s admission department, but whose actual discharge was delayed by more than 24 h. This study excluded patients who were discharged to other hospitals or referred to home hospitalization (those who continued to receive inpatient care at home).

### Variables

Data collection was based on information provided by the Admission and Economic Management Services of the hospital. We differentiated between variables related to length of stay, patient variables, and variables concerning the context of care. The total length of stay for these patients comprised two time periods: the length of the appropriate stay (from the admission date to the clinically fit for medical discharge date) and the length of the delayed stay (from the clinically fit for medical discharge date to the date that the patient left the hospital). In relation to the patient, the variables assessed were age, sex, Diagnosis-Related Group (DRG) and relative weight of the DRG to determine the complexity of the process. The relative DRG weight represents the average cost of caring for discharges in a specific DRG, regarding the average episode (weight = 1) (Yetano Laguna & López Arbeloa, 2010). Variables concerning the context of care included the type of admission (urgent or programmed), place of residence, *i.e.*, urban or rural (urban corresponding to residents in the same region as the hospital and with over 50,000 inhabitants and with a density of over 1,500 residents per km^2^, rural to other regions), and year of discharge. Two groups were established: the first group consisted of patients who died during the prolonged stay or BB period whereas the second group consisted of the remaining cases with delayed discharge for non-clinical reasons.

### Measures

Data analysis was performed using R 3.6.0 for Windows. For the descriptive analysis, proportions with their corresponding 95% confidence intervals (95% CI) were estimated for discrete variables. In the case of the continuous variables, the means, standard deviation (SD) and range were calculated. In order to compare the differences between deceased patients and the remaining patients who were BB, the Student’s *t*-test was applied for continuous quantitative variables and the Pearson’s chi-squared test was employed for categorical variables (*χ*^2^). An adjustment for multiple comparisons was performed by applying the Bonferroni correction, with a significance level of p 0.0015.

## Results

Out of a total of 3,015 patients in BB situations during the study period, 198 died during the period of prolonged stay, representing 6.57% (CI 95% [5.71–7.51]). The length of stay and the characteristics of these patients compared to the non-deceased patients are shown in Table 1.

**Table 1:** Comparison of deceased cases with bed blocking versus other cases of bed blocking: length of stay and patient characteristics. Cantabria (Northern Spain), 2007–2015.

		Deceased (*n* = 198)		Not Deceased (*n* = 2,817)		
		Mean [SD^a^], n (%)	Range, 95%CI^b^	Mean [SD^a^], n (%)	Range, 95%CI^b^	*p*-value
Length of total stay		27.45 [44.000]	(2–589)	28.59 [28.881]	(2–565)	0.606
Length of appropriate stay		16.76 [15.367]	(1–119)	21.49 [23.598]	(1–560)	0.005
Length of prolonged stay		10.69 [35.971]	(1–500)	7.10 [13.368]	(1–308)	0.002
Sex	Male	95 (47.98%)	(40.84–55.18)	1,349 (47.42%)	(45.57–49.27)	1.000
	Female	103 (52.02%)	(44.82–59.15)	1,468 (51.59%)	(49.74–53.45)	
Age (years)		77.27 [12.782]	(42–100)	77.39 [11.798]	(17–104)	0.893
DRG^c^ weight		3.65 [5.718]	(0.63–51.35)	3.77 [6.496]	(0.08–51.35)	0.805

**Notes.**

a SD, standard deviation.

b 95%CI, 95% confidence Interval.

c DRG, diagnosis-related group.

Regarding the length of stay in deceased patients, of the total of 5,436 days of length of total stay, 2,118 days corresponded to length of prolonged stay. The mean total stay duration was 27.45 days, with a median of 20 days and without significant differences with other non-deceased BB cases. The mean duration of appropriate stay was 16.76 days, with a median of 12.5 days, lower than the rest of the non-deceased cases, although without statistical significance (*p* = 0.005). In contrast, the prolonged stay had a mean duration of 10.69 days and a median duration of five days, in this case, this was longer compared to the rest of the non-deceased cases (*p* = 0.002), although this was not statistically significant.

Regarding the characteristics of the deceased BB patients, the most frequent DRG was simple pneumonia (8.58%, CI 95% [5.08–13.39]), although, overall, cases of neoplasia were recorded in 32.89% (CI 95% [26.3–39.8]) of the deceased patients. The mean age was 77.27 years (36.36% under 75) and the proportion of women was 52.02%. The mean DRG weight was 3.65, which is relatively high above the average episode (weight = 1). There were no statistically significant differences compared to the rest of the non-deceased cases.

The characteristics of the context of care and the comparison of deceased and non-deceased patients are shown in Table 2. In cases of patients who died during the prolonged stay period, 77.79% resided in urban areas and 95.45% were admitted urgently, with no differences compared to the rest of the patients. A total of 89.89% were admitted by a medical service, a significantly higher proportion than non-deceased patients (*p* < 0.001). From a total of fifteen services, those with the highest number of cases of deceased patients were Internal Medicine (46.97%), Oncology (22.73%) and Neurology (6.6%). The year with the highest number of deceased cases was 2007, although the decreasing progression was not as marked in the deceased group compared to the remaining patients.

**Table 2:** Comparison of deceased cases with bed blocking versus other cases of bed blocking: characteristics of the context of care. Cantabria (Northern Spain), 2007–2015.

		Deceased (*n* = 198)		Not Deceased (*n* = 2,817)		
		n (%)	95%CI^b^	n (%)	95%CI^b^	*p*-value
Place of residence	Rural	44 (22.22%)	(16.63–28.66)	634 (22.28%)	(20.77–23.86)	0.996
	Urban	154 (77.79%)	(71.34–83.36)	2,183 (76.73%)	(75.13–78.27)	
Type of hospitalization	Programmed	9 (4.54%)	(2.09–8.45)	202 (7.10%)	(6.18–8.11)	0.209
	Urgent	189 (95.45%)	(91.55–97.90)	2,615 (91.92%)	(90.85–92.89)	
Service	Medical	178 (89.89%)	(84.83–93.72)	1,976 (69.45%)	(67.73–71.14)	<0.001
	Surgical	20 (10.10%)	(6.28–15.17)	841 (29.56%)	(27.89–31.27)	
Year of discharge	2007	30 (15.15%)	(10.46–20.92)	342 (12.14%)	(10.96–13.40)	0.221
	2008	29 (14.65%)	(10.03–20.35)	416 (14.77%)	(13.48–16.13)	
	2009	12 (6.06%)	(3.17–10.35)	364 (12.92%)	(11.70–14.22)	
	2010	22 (11.11%)	(7.09–16.34)	340 (12.07%)	(10.89–13.33)	
	2011	29 (14.65%)	(10.03–20.35)	367 (12.90%)	(11.69–14.19)	
	2012	18 (9.09%)	(5.48–13.99)	279 (9.90%)	(8.82–11.07)	
	2013	23 (11.62%)	(7.51–16.92)	252 (8.95%)	(7.92–10.06)	
	2014	16 (8.08%)	(4.69–12.79)	211 (7.49%)	(6.54–8.52)	
	2015	19 (9.59%)	(5.88–14.58)	246 (8.65%)	(7.64–9.74)	

**Notes.**

a SD, standard deviation.

b 95%CI, 95% confidence interval.

## Discussion

In total, 3,015 patients were identified as having undergone a situation of bed blocking during the study period (Pellico-López et al., 2019). In total, 198 (6.57%) of the 3,015 patients died during their prolonged stay, a result that is in line with other studies that specifically address the problem of death during prolonged stay (Rosman et al., 2015), although another study found that one third of the patients who were waiting to be admitted in a post-acute care facility were palliative care patients (Soares et al., 2018).

In our sample of deceased patients with BB, the mean total stay duration was 27.45 days, which is lower than other studies that found a length of stay that was much greater than 30 days (Thomas & Ramcharam, 2010; Soares et al., 2018). In addition, the same bias can occur in our study because the proposed date of discharge was poorly recorded in the patient notes, making it difficult to determine the difference between appropriate and prolonged stay (Thomas & Ramcharam, 2010).

Comparing the length of stay between deceased patients and non-deceased bed blocked cases, there were no differences in the total length of stay, however, in the deceased patients, the length of stay that was considered adequate was shorter and the period of delay was longer. In patients qualifying for palliative care, clinicians can better identify when the therapeutic possibilities are exhausted and an acute hospitalization bed is no longer the appropriate resource for the patient, with a shorter length of stay (Soares et al., 2018). The bed-blocking situation in end-of-life patients represents much more than an inefficient use of the acute hospital bed, since it means that these patients do not receive the specific attention inherent to palliative care.

Regarding patient characteristics, the mean age of cases was 77.27 years, younger than in other similar studies. Over one third of patients were under 75 years of age, a result similar to studies that relate younger age to cases of oncology patients waiting for admission to a long-stay center for intermediate care (Soares et al., 2018).

The complexity among the deceased patients was relatively high, which may correspond to additional procedures and secondary diagnoses quantified in the DRG that increase complexity and prolong the length of stay, as is the case with the rest of the bed blocked patients (Holmas, Islam & Kjerstad, 2013). This result contradicts other studies on terminally ill patients, reporting a low intensity in the use of resources due to the exhaustion of therapeutic possibilities identified by the professionals in charge (Soares et al., 2018). Low complexity cases reflect BB cases in patients whose reason for admission has been described as family claudication or social problems without any medical trigger (Klop et al., 2018). Nevertheless, our study involves only one hospital and heterogeneity was observed between hospitals in end-of-life treatment intensity, suggesting the existence of condition-insensitive institutional norms (Barnato et al., 2015).

In our sample, 32.89% of patients were diagnosed with cancer. The most frequent DRG was simple pneumonia (Pellico-López et al., 2021), which could be due to factors such as advanced age, the presence of comorbidities and frailty or complications due to hospital infections (Rosman et al., 2015). In another study, dementia and stroke were the most prevalent conditions after cancer (Soares et al., 2018).

The deceased patients were more likely to be admitted to medical services as Oncology, which is similar to other studies (Thomas & Ramcharam, 2010; Soares et al., 2018). Oncology patients or patients affected by pneumonias - typical of patients with special fragility- may have been those suffering from a terminal illness who died while awaiting transfer to the most common discharge destination, which is the long-stay center that provides palliative care as well as functional recovery (Pellico-López et al., 2019).

Regarding the characteristics of the context of care, most deceased patients resided in the urban area in which both the hospital and the medium-long stay palliative care center are located. Our results are supported by the findings of a Spanish ecological study that demonstrated the relationship between the greater probability of dying in the hospital and residing in more urban areas with a greater availability of hospital beds (Jiménez-Puente & García Alegría, 2018). Our study reveals that patients died while waiting for the preferred place of discharge to become available at the end of their life.

Most of the cases of deceased patients were urgent admissions. For patients with advanced cancer, multiple emergency department visits are considered an indicator of poor quality of care. Emergency admissions during the last days of life can cause distress to the cancer patients and their caregivers. The reasons for emergency department visits in patients with advanced cancer are uncontrolled pain, altered mental status, dyspnea, fever, bleeding, infection, and neurological events (Delgado-Guay et al., 2015). In patients in an end-of-life situation, death has been observed to occur after admission to hospital, due to caregiver burnout. Caregiver distress was associated with greater hospital admissions for people with dementia living in the community (Afonso-Argilés et al., 2020; Guterman et al., 2019).

In patients at the end of life, the ratio of hospice beds in palliative care in the region during the study period was lower than recommended, even more so when there is a growing tendency to die in hospital (Jiménez-Puente & García Alegría, 2018). The increase in the number of medium-long stay beds has been shown to reduce hospital stays, representing a more efficient alternative to traditional hospitalization (Dahl et al., 2015). Demographic projections and trends in place of death point to an urgent need of service expansion. Developing services for end-of-life care for frail people with severe cognitive impairment in hospitals and care homes should be a priority (Perrels et al., 2014).

Our study period concluded in 2015, coinciding with the end of the economic recession in Spain. During this period, the efficient use of hospital services was of central importance, however, currently, the COVID pandemic requires additional physical and human resources at the end of life as part of the response to the pandemic (Chalk et al., 2021). The COVID pandemic has most likely worsened the situation of patients such as those described in our study. Patients whose management at the end of life is not possible at home will have found it more difficult to find an adequate palliative care resource in the hospital due to the internal reorganization that hospitals such as the one in our study area have undergone during the pandemic. Moreover, in elderly and especially frail patients, pneumonia has been a more frequent cause of death than ever before, and probably in a situation of isolation and loneliness due to the restrictions inherent to the pandemic. Therefore, the description of what happened with delayed discharge for non-clinical reasons in the years 2019–2022 would be an interesting line of future research.

### Strengths and limitations of this study

This study encompasses a nine-year period coinciding with the introduction of the dependency care system in Spain and the economic recession. All the wards of a high complexity hospital were included, located in a region with one of the most aged populations of Spain. The variables studied are based on data collected through the Hospital Records of Discharged Patients, which enables the systematic, homogeneous, and objective collection of variables at hospital discharge. Demographic, clinical (DRG), type of care or social context data were collected, which could be related to delayed discharge for non-clinical reasons. By using these Hospital Records of Discharged Patients, we ensured that data was systematically collected, and we were able to handle a large amount of data from an extensive period.

This study has several limitations. In the process of patient care, several other related variables exist, such as lack of social or family support or increasing their level of dependence for self-care. However, these data are not objectively reflected in the Hospital Records of Discharged Patients. Consequently, this kind of data is lost, requiring a dedicated review of the information recorded by the professionals in the patient’s clinical history.

## Conclusions

In people with terminal illness, clinicians can better identify when therapeutic possibilities are exhausted and acute hospitalization is not an adequate resource for their needs compared to the remainder of patients who are bed blocking. Living in an urban area with the availability of palliative care hospital beds is related to the decision to die in hospital. The number of patients in an end-of-life situation due to oncologic disease or frailty who die in an acute hospital bed shows the need to increase the availability of more appropriate resources such as hospice beds.

## Supplemental Information

10.7717/peerj.13596/supp-1 Supplemental Information 1 Dataset

10.7717/peerj.13596/supp-2 Supplemental Information 2 Codebook

## Additional Information and Declarations

### Data Availability

The following information was supplied regarding data availability: The data analysed to obtain our results and the codebook of the variables are available in the Supplementary Files.
