# Supplementary material for: Patient deaths during the period of prolonged stay in cases of delayed discharge for nonclinical reasons at a university hospital: a cross sectional study
Source: PeerJ. 2022 Jun 17;10:e13596. doi: 10.7717/peerj.13596 (PMC9208369; doi:10.7717/peerj.13596)
Supplement: Supplemental Information 2 [file peerj-10-13596-s002.docx]

| **Variables codification: “Patient deaths during the period of prolonged stay in cases of delayed discharge for nonclinical reasons at a university hospital: a cross sectional study”.** | | |
| --- | --- | --- |
| **Variable** | **Label** | **Codification** |
| LOS | Length of total stay | [DATE_ED]-[ DATE_ADM]  Days (00) |
| LAS | Length of appropriate stay | [DATE_DISCH]-[ DATE_ADM]  Days (00) |
| LPS | Length of prolonged stay | [DATE_ ED]-[ DATE_ DISCH]  Days (00) |
| AGE | Age | Years (00.00) |
| SEX | Sex | 0= male; 1= female |
| DRG_WEIGHT | DRG weight | Quantitative (00.0000) |
| RURAL_URB | Place of residence | 0= urban; 1=rural |
| ADMISS | Type of hospitalization | 0=programmed; 1=urgent |
| MED_SURG | Service | 0=medical; 1=surgical |
| YEAR_DISCH | Year of medical discharge | Qualitative (2007, 208, 2009, 2010, 2011, 2012, 2013, 2014, 2015) |
| DEATH | Deceased with bed blocking versus other cases of bed blocking | 0= other cases; 1=deceased |
